# Overexpression of Her-2 in Biopsy-Proven Urothelial Carcinoma Patients From Pakistan

**DOI:** 10.7759/cureus.23739

**Published:** 2022-04-01

**Authors:** Anila Chughtai, Ghazi Zafar, Fatima Khalid, Sameen Afzal, Beenish Usman, Akhtar S Chughtai

**Affiliations:** 1 Histopathology, Chughtai Institute of Pathology, Lahore, PAK

**Keywords:** urinary bladder carcinoma, urothelial carcinoma, immunohistochemistry staining, her2 overexpression, her-2

## Abstract

Background

Bladder cancer is a common urological cancer. Her-2 gene is a proto-oncogene which is present in 17q12 chromosomal region. Her-2 overexpression has been seen in breast, gastric and ovarian cancers. The purpose of this study was to evaluate the incidence of overexpression of Her-2 in patients with urothelial carcinoma diagnosed by histopathology in the Pakistani population.

Methods

This is a cross-sectional observational study conducted on the biopsy samples of patients diagnosed with urothelial carcinoma in Histopathology Department of Chughtai Institute of Pathology from 15 September 2018 to 15 March 2021. The immunohistochemical analysis was done on serial sections using immune-enzymatic soluble complex method. The antibody used was Her-2 polyclonal antibodies from DAKO (Agilent, Santa Clara, USA). Her-2 scoring was done according to the College of American Pathologists (CAP) guidelines for reporting Her-2 overexpression in breast cancer.

Results

A total of 140 cases of urothelial cancer were included in the study. About 83.57% (n=117) of cases were males, and 16.42% (n=23) were females. Positive Her-2 staining was observed in 38/140 (27.15%) cases. A significant association was seen between Her-2 staining and muscle invasion (p-value=0.0001).

Conclusion

Our study shows that Her-2 overexpression is seen in a number of patients with urothelial carcinoma, especially in patients with muscle invasion. These patients may benefit from targeted therapy against the Her-2 gene. Her-2 overexpression evaluation should be considered in such patients.

## Introduction

Bladder cancer is a common urological cancer with a high recurrence rate. The most common type is urothelial carcinoma (UC). It is three times more common in men as compared to women. Mostly, it is non-muscle invasive at diagnosis but has a high recurrence rate with nearly 70% of patients having recurrence within five years. The outcome is poorer for those with advanced disease, with five-year survival being <15%. Most patients are elderly when the tumor is diagnosed (median: 73.2 years). More than 90% of bladder cancers are urothelial carcinoma. Although it may originate anywhere in the urinary tract, the most common site is the urinary bladder. Tobacco smoking, radiation therapy, occupational exposure to aromatic amines, consumption of arsenic-laced water and chemotherapeutic drugs such as alkylating agents are the common risk factors [1]. Despite multidisciplinary advancement in treatment, the combined five-year survival rate for all stages is 77% [2].

Her-2 gene is a proto-oncogene which is present in 17q12 chromosomal region. It has been shown that in cases of breast cancer, the overexpression of this receptor was associated with poor prognosis and increased incidence of disease recurrence. Her-2 overexpression has also been seen in gastric and ovarian cancers [3,4]. Few studies have shown that similar overexpression is also seen in some cases of urothelial carcinoma [5]. Trastuzumab, a monoclonal antibody directed against the Her-2 gene, was one of the first molecular targeted drugs to be developed. Over the last decade, trastuzumab has revolutionized the treatment of Her-2-positive breast cancer and tremendously improved its outcomes. On the basis of these findings, it is suggested that patients with urothelial carcinoma should have their tumor tested for Her-2 amplification at the time of the initial diagnosis as potential target for trastuzumab.

Different studies show variable expression of Her-2 in different populations. There are no published studies on the Pakistani population to the best of our knowledge. The aim of our study is to assess Her-2 overexpression by immunohistochemical (IHC) method in our population with urothelial carcinoma (UC) so that these patients can benefit from the targeted drugs.

## Materials and methods

Study design

We carried out an observational study using nonprobability consecutive sampling technique at Chughtai Institute of Pathology, Lahore.

Duration

The study was carried out from 15 September 2018 to 15 March 2021.

Inclusion criteria

All histologically diagnosed cases of urothelial carcinoma, between the ages of 30 to 70 years of both genders, were included.

Exclusion criteria

Autolyzed samples were excluded from the study.

Data collection

Prior to the start of the study, ethical approval was obtained from Chughtai Institute of Pathology (CIP) Institutional Review Board (Letter No. CIP/IRB/1102). The age and gender of patients were noted. Histological sections were prepared from paraffin blocks and stained with hematoxylin-eosin stains followed by immunohistochemical analysis using immune-enzymatic soluble complex method. Her-2 polyclonal antibodies by DAKO (Agilent, Santa Clara, USA) were used. Her-2 scoring was done according to the College of American Pathologists (CAP) guidelines for breast cancer as 3+ score (complete, intense, circumferential membrane staining in >10% of invasive tumor cells) was considered positive. All cases with 0 and 1+ Her-2 scoring were considered negative. Cases with 2+ (equivocal) Her-2 score were also considered negative as no further fluorescence in situ hybridization (FISH) testing was done on these cases.

Statistical analysis

Data were analyzed using SPSS version 20 (IBM, New York, USA). The mean and standard deviation were calculated for quantitative variable. The frequency and percentages were calculated for qualitative variables. Effect modifiers, gender, histological grade and invasion, were stratified. Poststratification chi-square test was done. p-value less than or equal to 0.05 was taken as significant.

## Results

**Figure 1:**
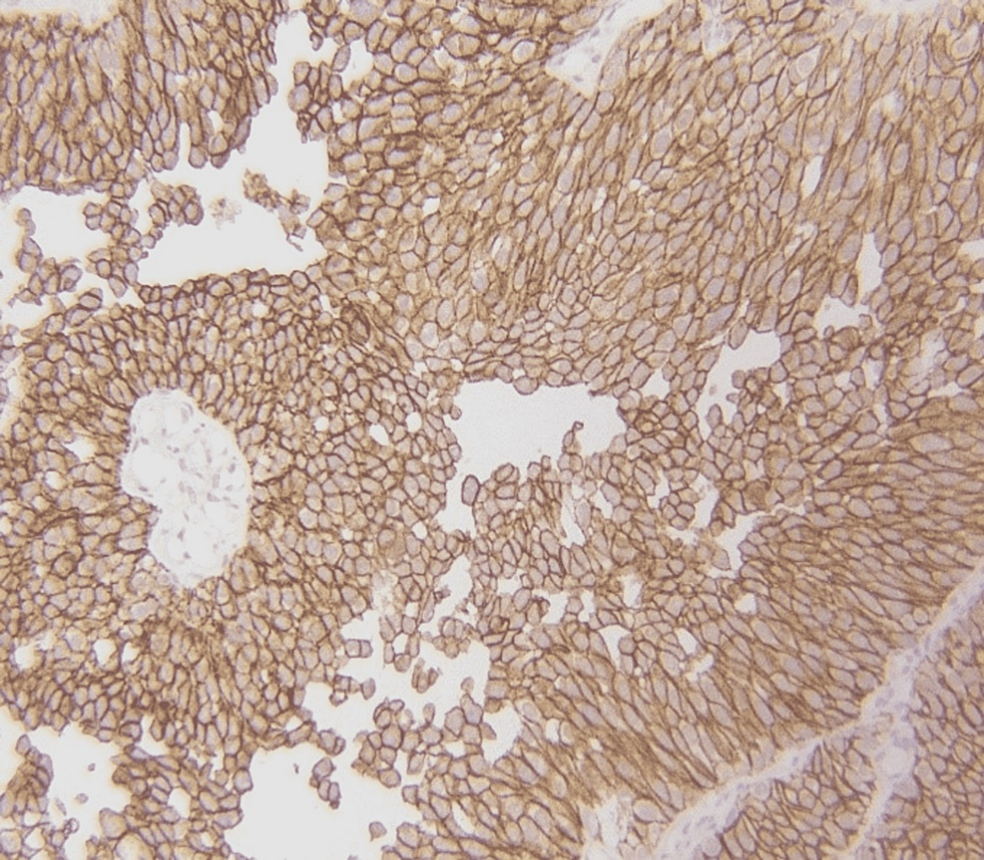
Positive (3+ Her-2 scoring)

A total of 140 cases fulfilling the selection criteria were enrolled in the study. The mean age of the patients was 61.05±13.32 years. A majority of the patients were male (83.57%). Most of the tumors were high grade (57.15%) and non-muscle invasive (80.7%). The frequency of Her-2 immunohistochemical stain positivity (3+) (Figure 1) was recorded in 27.15% (Table 1). 

**Table 1:** Summary of results (n=140)

Age	
Age (years)	61.05±13.32
Frequency and percentage of gender	
Male	117 (83.57%)
Female	23 (16.42%)
Frequency and percentage of patients according to overexpression of HER-2	
Positive	38 (27.15%)
Negative	102 (72.85%)
Frequency and percentage of patients according to grade of tumor	
Low grade	60 (42.85%)
High grade	80 (57.15%)
Frequency and percentage of patients according to invasion of muscle	
Muscle invasive	27 (19.3%)
Non-muscle invasive	113 (80.7%)

No significant association was noted between Her-2 overexpression and gender or tumor grade. However, when stratified for muscle invasiveness, we found a significant correlation (p-value=0.0001) (Table 2). The majority of the tumors expressing HER-2 belonged to the muscle-invasive subgroup.

**Table 2:** Stratification of overexpression of HER-2 with respect to different variables

	Overexpression of HER-2		p-value
	Yes	No	
Stratification according to gender			
Male	33 (28.21%)	84 (71.79%)	0.523
Female	5 (21.74%)	18 (78.26%)	
Stratification according to grade			
Low grade	16 (26.66%)	44 (73.33%)	0.912626
High grade	22 (27.5%)	58 (72.5%)	
Stratification of overexpression of HER-2 with respect to muscle invasion			
Muscle invasive	21 (77.8%)	6 (22.2%)	0.0001
Non-muscle invasive	17 (15%)	96 (85%)	

## Discussion

Bladder cancer is a common malignancy. The five-year survival rate is only about 10%-15%, especially in locally advanced and metastatic diseases. Most of the patients with urothelial carcinoma inevitably succumb to this disease despite aggressive chemotherapy [6]. Recently, significant work has been done in the evaluation of different possible therapeutic targets in patients with urothelial carcinoma. Her-2/ERBB2 gene is one of those targets of interest which is largely been assessed for its role in the treatment of urothelial carcinoma. Her-2 belongs to the epidermal growth factor receptor (EGFR) family. Anti-Her-2 drug has emerged as a promising targeted drug in these tumors with impressive improvements in overall survival [7]. The need for this study was that only a few studies have recently been done assessing Her-2 overexpression in urothelial carcinoma. Although studies have accessed Her-2 overexpression in cases of gastric and breast carcinomas to the best of our knowledge, no study has evaluated Her-2 overexpression in patients with urothelial carcinoma in our country even though Pakistan has a high incidence of urothelial carcinoma of the urinary bladder.

The mean age of the patients in our study was 61.05±13.32 years. The median age as noted in the National Comprehensive Cancer Network (NCCN) guidelines was 73 years [8]. This is in line with our findings that urothelial carcinoma is rare before the fifth decade of life. The pathology is more common in men as compared to women. In our study of 140 patients, 117 (83.57%) were male and 23 (16.4%) were females. Siegel et al. noted a male preponderance in their study as well [9]. Regarding grade, 60 (42.85%) patients have low grade, while 80 (57.15%) patients have high grade. Andreassen et al. found similar findings while reviewing data from over 33,000 patients who were diagnosed with urothelial carcinoma of the bladder between 1981 and 2014 [10]. Different studies have reported overexpression of Her-2 from 12% to 71% in urothelial cancers. It has also been reported that there is a statistically significant difference in Her-2 overexpression detected by IHC in high-grade urothelial carcinoma compared with low grade [11]. In our study, we observed Her-2 overexpression in 27.15% of the specimen; however, we found no significant difference in overexpression of Her-2 when grade of tumor was considered. When stratified for age and gender, the relation of Her-2 overexpression to either was not significant. Nedjadi et al. in their study published in 2016 found that there was no significant relation of age, gender or muscle invasiveness with Her-2 overexpression [12].

Two different subtypes of bladder carcinoma are recognized: muscle invasive and non-muscle invasive. The muscle-invasive subtype is associated with a poorer outcome, while recurrences are more commonly seen in the non-muscle-invasive subtype. Most of the studies evaluating the Her-2 overexpression in bladder carcinoma have been done on muscle-invasive bladder carcinoma. Variation in the incidence of overexpression of HER2 has been seen in different studies [13]. Bellmunt et al. reported variation of overexpression among different populations [14]. Reports of Her-2 status in non-muscle-invasive bladder carcinoma (NMIBC) are limited. A few studies have shown Her-2 protein overexpression in 4%-12% of NMIBCs. Although not intentional, a majority of cases in our study are of non-muscle-invasive subtype. Despite this, the expression of HER2 protein was not significant in the non-muscle-invasive subtype. In a recent study from Indian Subcontinent, Agrawal et al. found HER2 2/3+ expression (46%) in patients with urothelial carcinoma [15]. Similarly, we also found Her-2 expression in 27.15% of urothelial carcinoma cases of which the majority belonged to the muscle-invasive subtype. The percentage of Her-2 expression in the non-muscle-invasive subtype in our study (15%) was similar to that noted by Agrawal et al. [15].

The main limitation of our study is that it had a small sample size and cases with equivocal 2+ Her-2 score were not further assessed by fluorescence in situ hybridization (FISH) technique and were considered negative. Since incidence of urothelial carcinoma is increasing in Pakistan and the fact that patients with an overexpression are candidates for potential targeted therapy, a more extensive assessment of Her-2 overexpression in such patients is needed in our population. Our study does point out that the pattern of overexpression of Her-2 appears to be similar to the one reported by researchers in other parts of the world [8-10,12,15].

## Conclusions

We conclude that a significant percentage of urothelial carcinomas of the urinary bladder in patients from Pakistan show Her-2 overexpression raising the strong possibility of them being a potential target for anti-Her-2 therapy. This is more common in the muscle-invasive subtype of urothelial carcinoma. Therefore, patients with urothelial carcinomas should be considered for routine testing for Her-2 overexpression.
